# Racial Disparities in Health Risk Indicators Reported by Alabamians Diagnosed with COPD

**DOI:** 10.3390/ijerph18189662

**Published:** 2021-09-14

**Authors:** Michael Stellefson, Min-Qi Wang, Caitlin Kinder

**Affiliations:** 1 Department of Health Science, The University of Alabama, Tuscaloosa, AL 35487, USA; 2 Department of Behavioral and Community Health, University of Maryland, College Park, MD 20742, USA; mqw@umd.edu; 3 College of Education, The University of Alabama, Tuscaloosa, AL 35487, USA; cmkinder@crimson.ua.edu

**Keywords:** BRFSS, COPD, health risks, latent class modeling, Alabama, comorbidity, healthcare access

## Abstract

Chronic Obstructive Pulmonary Disease (COPD) is a growing public health problem in the southern United States, particularly in Alabama. However, very little is known about specific health risk factors disproportionately impacting Alabamians with COPD. We conducted a latent class analysis of 2015–2019 Behavioral Risk Factor Surveillance System data from 4057 Alabamians with COPD (White = 2947, Black = 873, Other = 237). Eighteen risk indicators were examined across three health-related domains: (1) comorbidities, (2) limited healthcare access, and (3) substance use/abuse. Racial disparities between Black and white Alabamians with COPD were assessed using configural similarity analysis. Findings showed that almost one-third (31%) of Alabamians with COPD were in the high-risk class for eight comorbidities, and nearly one-half (48.88%) belonged to the high-risk class for limited healthcare access. Black Alabamians with COPD who did not have health insurance were much more likely to be at high risk for limited healthcare access (94.44%) when compared to their counterparts with insurance (5.56%), *χ*^2^(*df* = 2) = 1389.94, *p* < 0.0001. Furthermore, the proportion of high-risk, uninsured Black Alabamians with COPD (94.44%) substantially exceeded the percentage of high-risk, uninsured white Alabamians with COPD (59.70%). Most Alabamians with COPD (82.97%) were at low risk for substance use/abuse. Future research should explore new mechanisms for facilitating better healthcare access among high-risk Alabamians living with COPD and other prevalent comorbidities. Greater attention should be focused on Black Alabamians with COPD who cannot afford adequate health insurance.

## 1. Introduction

Adults living with Chronic Obstructive Pulmonary Disease (COPD) experience dyspnea (i.e., shortness of breath) and excessive mucus production, perpetuating a vicious cycle of symptomatic living that adversely impacts physical and mental health [1,2,3]. Nevertheless, COPD is manageable if patients are aware of their susceptibility to health risks. While combustible cigarette smoking is the most critical risk factor for COPD exacerbations, the COPD National Action Plan [4] emphasizes identifying the range of individual and health system risk factors associated with poor COPD-related outcomes across different regions of the United States (U.S). States in the southern U.S. report some of the worst COPD outcomes [5]. For example, Alabama has the second-highest state prevalence of COPD in the entire U.S., with nearly 10% of the state’s population (i.e., close to 400,000 Alabamians) likely to be living with COPD [6]. In 2020, chronic lower respiratory disease was the third leading cause of death in Alabama [7]. Approximately 53 out of every 100,000 Alabamians die from COPD each year [8]. Annually, the cost of treatment for COPD in Alabama is approximately $543 million [9]. 

As of 2015, Alabama was one of seven U.S. states in the highest quartile for the following statistics: state-specific age-adjusted prevalence of COPD among adults aged ≥18 years, hospitalization rates among Medicare enrollees aged ≥65 years, and age-adjusted death rates [6]. In the most recent U.S. state assessment of COPD burden [5], Alabama demonstrated a higher than average prevalence and generally severe burden of COPD on most dimensions of morbidity and mortality. Furthermore, Alabama received abysmal grades ranging from “4” to “5” on a scale of “1” being the best possible score and “5” being the worst possible score for the following metrics: age-adjusted mortality rate (Grade = 5), percentage of adults with COPD (Grade = 5), the average burden of morbidity and mortality (Grade = 4), and the number of COPD patients enrolled in pulmonary rehabilitation (Grade = 4) [5]. Disparities in COPD diagnoses are also evident across the state based on income level. Alabamians who make less than $15,000 per year are almost five times more likely to be diagnosed with COPD than those who make over $50,000 [9]. In addition, compared to their counterparts without COPD, Alabamians with COPD are more likely to report that cost was an obstacle to accessing healthcare [10]. 

In addition to these health and healthcare disparities based on income, the Alabama state prevalence of COPD is higher than national averages across different racial groups. Across the U.S., COPD affects approximately 6.1% of Black Americans compared to 6.3% in non-Hispanic white Americans [11,12]. In Alabama, these percentages are higher, with 11.1% of white and 7.0% of Blacks reporting a diagnosis of COPD [13]. In particular, Black Americans are at greater risk for undiagnosed COPD than whites [14] and thus represent the paradigm of a group of people at risk for health disparity and health inequity [15,16]. Data from 1980 to 2000 show that death rates caused by COPD rose by 87% in black individuals compared to only 67% in non-Hispanic white individuals [12]. Black Americans with COPD also experience higher hospitalization and emergency room visits than whites, with fewer Blacks with COPD reporting regularly scheduled physician office visits [17]. While general health status is similar for Blacks and Caucasians with COPD who do not experience exacerbations, it worsens for Blacks who report exacerbations, particularly those requiring hospitalization [18]. 

Numerous factors are thought to disproportionately impact COPD treatment and management for Black Americans [19]. Prolonged cigarette smoking, combined with abnormal body mass index (BMI) and comorbidities, can increase health risks associated with COPD [20]. Black Americans who experience COPD exacerbations and are treated at Veterans Affairs hospitals are more likely to be admitted to the Intensive Care Unit (ICU) for mechanical ventilation than non-Hispanic whites, supporting the notion that Blacks may be more prone to more severe COPD exacerbations [12]. Other factors such as racial residential segregation are also associated with more significant COPD morbidity among urban Blacks, suggesting that racial segregation may also play a role in explaining some existing health inequities [21]. However, even after accounting for individual and neighborhood SES factors, Black individuals showed increased risks for severe exacerbations and persistently worse computerized tomography (CT) scans of the lungs [22]. One small cohort of Alabamians with COPD recruited from a pulmonary clinic and research center more frequently cited lack of transportation, food insecurity, low monthly incomes, and higher neighborhood discord than whites and those representing other ethnicities [23]. 

The precise causes and pathophysiologic significance of these racial differences are currently unknown [24]. Beyond existing social and structural inequalities, there is a paucity of population-based studies examining differences in existing health risk factors, specifically between Black Americans and other Americans living with COPD. Although Black Alabamians with COPD may be more socially vulnerable than other racial groups with COPD [23], almost no research has examined the potential for racial disparities in other highly relevant health indicators, such as comorbidities, substance use and abuse, and access to healthcare services. Therefore, we examined the prevalence of these health risk factors in Alabamians with COPD, focusing on existing disparities between Black and white Alabamians living with COPD. We hypothesized the following:

1. Most Alabamians with COPD would be classified at high risk on the three health-related domains (i.e., comorbidities, substance use and abuse, and access to healthcare services); and
2. The prevalence of health indicators making up each latent domain would differ according to race when most Alabamians with COPD were at intermediate or high (i.e., elevated) risk.

## 2. Materials and Methods

The Behavioral Risk Factor Surveillance System (BRFSS) is a population-based, random-digit-dialed telephone survey that monitors access to healthcare, health conditions, and behavioral risks contributing to the leading causes of disease among U.S. adults 18 years or older [25]. We conducted a cross-sectional analysis of pooled 2015–2019 AL BRFSS data collected from respondents (*n* = 4057) who answered “Yes” to the question, “Has a health professional EVER told you that you have COPD, emphysema, or chronic bronchitis?” The Office for Research Compliance at the university where the study took place approves investigator use and secondary analysis of specific public datasets without application to the Institutional Review Board (IRB). In addition, the human research protection program at the university approves the use of public BRFSS data without investigators seeking formal IRB approval.

**Figure 1:**
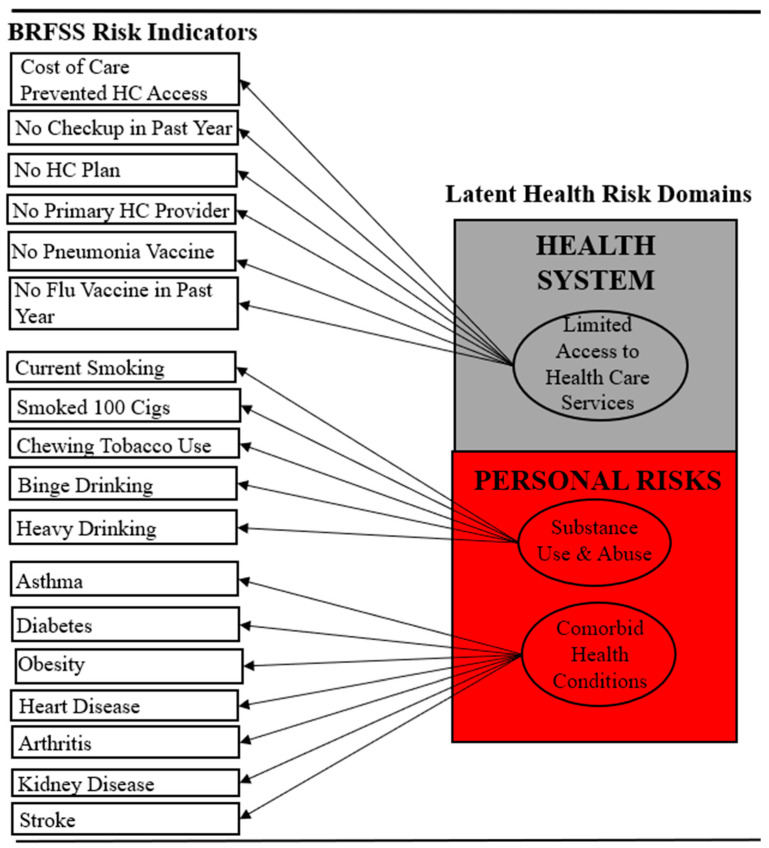
LCM of BRFSS risk indicator data collected from Alabamians diagnosed with COPD.

We developed a list of ≈18 BRFSS risk indicators potentially linked to health disparities disproportionately affecting Black Americans with COPD [15,16,22]: (1) comorbidities; (2) substance use and abuse; and (3) limited access to healthcare. Rather than using summated indices to assess risk within each of these domains, we used latent class modeling (LCM), which is a data analysis technique that collapses numerous discrete indicators into a set of parsimonious latent classes (i.e., risk categories) and estimates the probability that a respondent belongs to a mutually exclusive class based on their pattern of responses on a set of variables. LCM was ideal for this study due to the need for untangling complex relationships between health risk indicators. We estimated three sets of discrete latent class models with eight BRFSS indicators (i.e., asthma, diabetes, obesity, cardiovascular disease, arthritis, stroke, depression, kidney disease) for the comorbidity domain, six BRFSS indicators (i.e., no health insurance, no primary healthcare provider, cost prevented medical care, no check-up in the past year, no flu shot in the past year, no pneumonia vaccine) for the limited healthcare access domain, and five BRFSS indicators (i.e., smoked at least 100 cigarettes over a lifetime, current smoker, current use of chewing tobacco, binge drinking, heavy drinking) for the substance use and abuse domain. Figure 1 lists all risk indicators that were evaluated under the “BRFSS Indicators” column. For each LCM, we estimated results for the entire sample of Alabamians with COPD and then separate LCMs for Black versus white Alabamians with COPD.

LCM empirically investigates whether the assumption about the relationship between the latent variable and the frequencies of reported risk factors is acceptable. The analysis enables researchers to identify mutually exclusive groups that adequately describe the dispersion of observations in the n-way contingency table of discrete indicators. In other words, the response pattern on a set of variables is the unit of analysis that classifies subgroups into mutually exclusive types of latent risk classes (e.g., high, intermediate, or low) [26,27]. For example, a class 1 membership may indicate that most class 1 respondents show low levels of current smoking, heavy drinking, or binge drinking, and we may classify this membership as a “low risk” group for substance use and abuse. In contrast, a class 3 membership may detect that most respondents in class 3 report smoking, heavy drinking, or binge drinking; we may classify this membership as the “high risk” group for substance use and abuse. We may also find a class membership between 1 and 3, classified as an “intermediate” risk group. LCM uses an iterative approach in which models are estimated in succession and compared using model fit indices.

To determine the most parsimonious LCM for each health risk domain, we sequentially fit and compared models [28] using Akaike’s information criterion, Bayesian information criterion (BIC), sample-size adjusted BIC, model interpretability, and classification accuracy using the entropy value. In addition, we followed guidelines from prior research [29] recommending that the smallest BIC value is the best empirical justification for the number of classes to retain. 

To augment results from the LCM analyses, we utilized configural similarity analysis to assess the potential for racial disparities in latent risk structures across two racial groups (Black vs. white). Configural similarity is the first step to assess similarity across subgroups for a multi-group LCM [30]. We evaluated two separate LCMs (white vs. Black) to assess the configural similarity of each domain. This analysis described whether or not the two racial groups, when viewed in isolation of each other (i.e., white vs. Black), contained similar latent structures on each risk indicator [31]. LCM analyses were conducted in Mplus (v7.3) to accommodate the complex sampling design of BRFSS [32]. Subsequent 2 (race) × 3 (class) chi-square analyses were conducted to examine differences within the low, intermediate, and high-risk classes based on select races relevant to the purpose of the study (white vs. Black).

## 3. Results

The LCM for the entire sample (*n* = 4057) suggested the best fit was for three latent classes (i.e., low, intermediate, and high risk) in comorbidity, substance use/abuse, and limited healthcare access domains. 

### 3.1. Comorbidities

**Figure 2:**
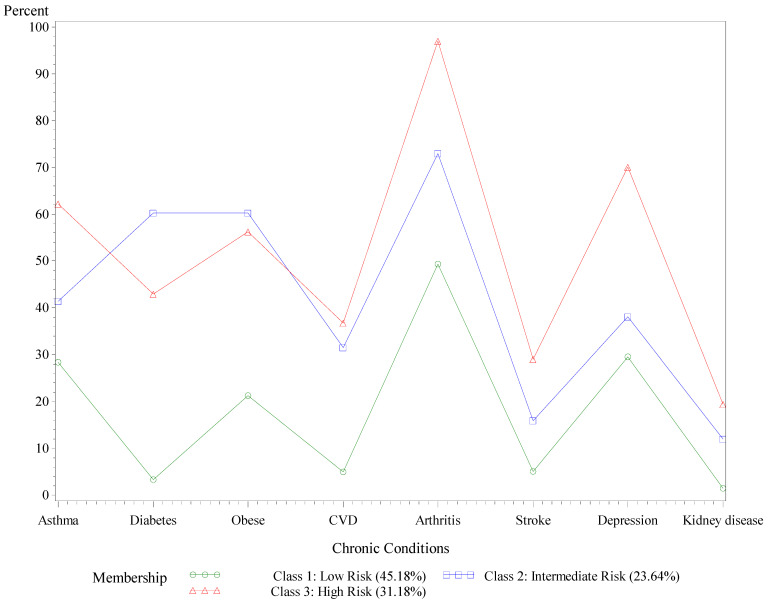
Probabilities of chronic health condition by latent class, AL adults with COPD, BRFSS, 2015–2019. Comorbid health conditions are defined as having ever been told by a health professional that respondents had the condition of asthma, diabetes, obesity, cardiovascular disease (CVD), arthritis, stroke, depressive symptoms, or kidney disease.

About 31% of Alabamians with COPD reported being in the high-risk comorbidity class for the eight health conditions (see Figure 2). Over 90% of respondents in the high-risk class reported arthritis (97.57%), around 70% reported symptoms of depression, and more than half reported asthma (62.21%) and obesity (56.29%). About 23% of Alabamians with COPD belonged to an intermediate risk comorbidity class: 60.23% reported diabetes, reported 60.23% obesity, and over 70% reported arthritis. In the remaining 45.18% at low risk for comorbidities, 28.34% reported asthma, 49.34% reported arthritis, and 29.53% reported symptoms of depression.

### 3.2. Substance Use Behaviors

**Figure 3:**
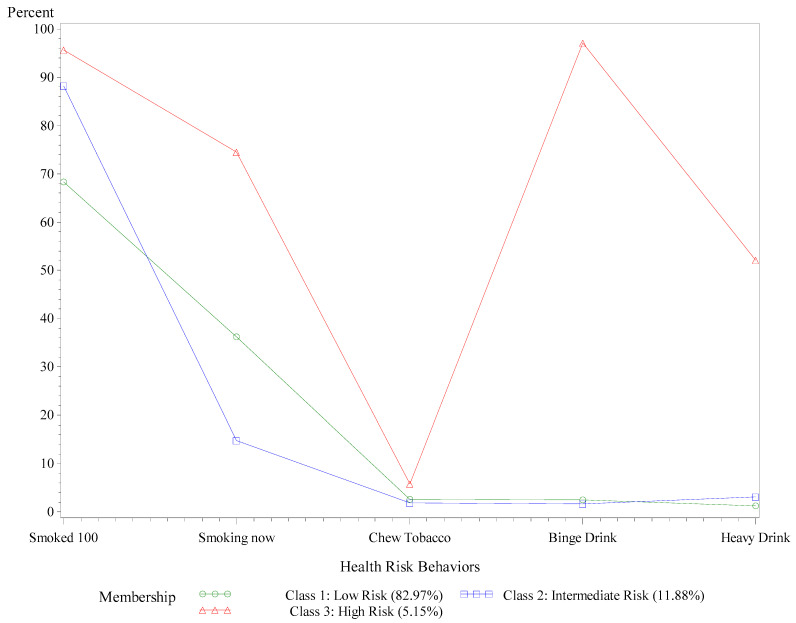
Prevalence probabilities of substance use and abuse behaviors by latent class, AL adults with COPD, BRFSS, 2015–2019. Binge drinking is defined as ≥five drinks for men or ≥four drinks for women on one occasion during the past 30 days. Heavy drinking is defined as men having >14 drinks per week and women having >7 drinks per week. Behaviors refer to the past 30 days.

Contrary to our hypothesis, only about 5% (5.15%) of Alabamians with COPD classified into the high-risk class for the five substance use behaviors (see Figure 3). Of these high-risk substance users, 74.50% smoked every day, 52.12% were heavy drinkers, and 97.13% reported binge drinking in the past 30 days. Slightly more than 10% (11.88%) of Alabamians with COPD made up an intermediate risk class. Among respondents at intermediate risk, 88.24% had smoked at least 100 cigarettes throughout their lifetime, but only 14.76% were current smokers. A small percentage of respondents in the intermediate-risk class chewed tobacco (1.9%), binge drank (1.66%), or drank heavily (3.14%). In the remaining 82.97% of AL adults with COPD in the low-risk class, 68.29% had smoked at least 100 cigarettes for their lifetime, and 36.23% were current smokers. Similar to the intermediate class, less than 3.0% of those in the low-risk class chewed tobacco, binge drank, or drank heavily.

### 3.3. Healthcare Access

**Figure 4:**
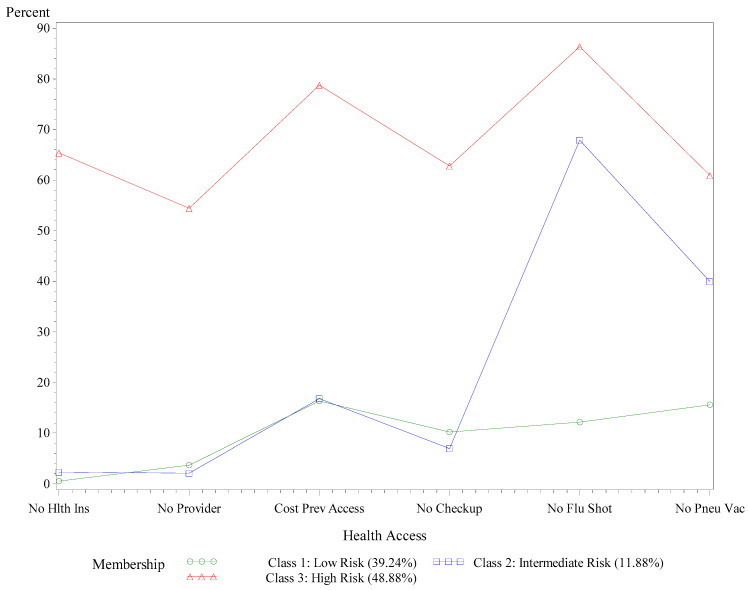
Probabilities of limited healthcare access by latent class, AL adults with COPD, BRFSS, 2015–2019. No Hlth Ins is defined as a response of “No” healthcare coverage, including health insurance, prepaid plans such as HMOs, or government plans such as Medicare or the Indian Health Service. No Provider is defined as a response of “No” when asked if there was a person the respondent could identify as a personal doctor or healthcare provider. Cost Prev Access is defined as a response of “Yes” when asked if there was a time in the past 12 months when the respondent needed to see a doctor but could not because of cost. No check-up is defined as not visiting a doctor for a routine check-up within the past 12 months. No flu shot is defined as not receiving a flu shot or flu vaccine during the past 12 months. No Pneu Vac is defined as never receiving a pneumococcal vaccine.

In support of our hypothesis, we found that almost one-half of Alabamians with COPD (48.88%) belonged to the high-risk class for limited healthcare access (see Figure 4). Among those at high risk for limited access to care, 65.35% reported having no healthcare plan, 54.47% did not have a primary healthcare provider, 78.79% could not afford medical costs, 62.74% had a routine check-up more than one year ago, 86.36% did not get their flu shot in the past year, and 60.89% had never received their pneumonia vaccination. Among the 11% of respondents classified into the intermediate-risk class, 67.89% did not get their flu shot in the past year, and 39.91% never received their pneumonia vaccination. Of the 39.24% at low risk for inadequate healthcare access, less than 20% reported difficulty accessing healthcare services, yet 12.19% did not get their flu shot in the past year, and 15.60% never received their pneumonia vaccination. 

### 3.4. Racial Disparities in Risk Classes for Comorbidity and Limited Healthcare Access

The majority of Alabamians with COPD were at either “high” or “intermediate” risk for comorbidities (68.82%) and limited healthcare access (60.76%). Therefore, to assess the configural similarity of these elevated health and healthcare risk domains, we examined latent classes within these domains according to race (i.e., Black vs. white). As with the entire sample, the two separate LCMs (i.e., one LCM including only white respondents and one LCM including only Black respondents) suggested that the three risk classes (i.e., low, intermediate, and high risk) fit the data best in both models. 

Table 1 lists chi-square results describing statistically significant differences in the frequencies of white and Black participants assigned to each latent risk class for each comorbidity indicator. White Alabamians who reported asthma in addition to COPD were more likely to be at high risk for comorbidities (63.27%) compared to those without asthma (36.73%), *χ*^2^(*df* = 2) = 488.62, *p* < 0.0001. Conversely, Black Alabamians who reported asthma with COPD were less likely to be at high risk for comorbidities (25%) than those without asthma (75%), *χ*^2^(*df* = 2) = 101.62, *p* < 0.0001. Obese white Alabamians with COPD were more likely to be at high risk for comorbidities (57.56%) than whites who were not obese (42.44%), *χ*^2^(*df* = 2) = 420.49, *p* < 0.0001. In contrast, obese Black Alabamians with COPD were much less likely to be at high risk for comorbidities (7.69%) compared to non-obese respondents (92.31%), *χ*^2^(*df* = 2) = 204.64, *p* < 0.0001. 

Black Alabamians with COPD and cardiovascular disease were far more likely to be at high risk for comorbidities (82.14%) than those without cardiovascular disease (17.86%), *χ*^2^(*df* = 2) = 155.18, *p* < 0.0001. On the other hand, white Alabamians with cardiovascular disease were less likely to be at high comorbidity risk (35.67%) compared to those without cardiovascular disease (64.33%), *χ*^2^(*df* = 2) = 405.09, *p* < 0.0001. Additionally, Black Alabamians diagnosed with COPD and chronic kidney disease were considerably more likely to be at high risk for comorbidities than Black patients without kidney disease, *χ*^2^(*df* = 2) = 300, *p* < 0.0001. White Alabamians with COPD and kidney disease were less likely to be at high comorbidity risk (17.60%) when compared to white COPD patients without kidney disease, *χ*^2^(*df* = 2) = 204.16, *p* < 0.0001.

Table 2 describes statistically significant chi-square analyses examining the frequency of white and Black participants assigned to each risk class based on limited healthcare access indicators. Black Alabamians with COPD who did not have health insurance were much more likely to be at high risk for limited healthcare access (94.44%) when compared to their counterparts with insurance (5.56%), *χ*^2^(*df* = 2) = 1389.94, *p* < 0.0001. Furthermore, this proportion of high-risk, uninsured Black Alabamians with COPD (94.44%) substantially exceeded the percentage of high-risk, uninsured white Alabamians with COPD (59.70%). White Alabamians with COPD who did not report a check-up in the past year (68.09%) were more likely to be at high risk for limited healthcare access than whites who had gone to the doctor for their annual check-up (31.91%), *χ*^2^(*df* = 2) = 806.23, *p* < 0.0001. Conversely, Black Alabamians with COPD who had their annual check-up were slightly more likely to be at high risk for limited healthcare access (52.81%) than those without an annual check-up (47.19%), *χ*^2^(*df* = 2) = 188.98, *p* < 0.0001.

White Alabamians with COPD who received their annual flu shot were more likely to be at low risk for limited healthcare access than their counterparts who did not get their flu shot, *χ*^2^(*df* = 2) = 2113.28, *p* < 0.0001. In contrast, Black Alabamians with COPD who did not report receiving an annual flu shot (97.31%) were much more likely to be at low risk for limited healthcare access compared to those who received their flu shot (2.87%), *χ*^2^(*df* = 2) = 233.54, *p* < 0.0001. Similarly, Black Alabamians with COPD who never received their pneumococcal vaccination (90.70%) were also more likely to be at low risk for limited healthcare access when compared to Black Alabamians with COPD who got the pneumococcal vaccine (9.30%), *χ*^2^(*df* = 2) = 233.54, *p* < 0.0001. As with the flu shot, White Alabamians with COPD who received their annual flu vaccine were dramatically more likely to be at low risk for limited healthcare access, *χ*^2^(*df* = 2) = 2113.28, *p* < 0.0001.

## 4. Discussion

COPD is a growing public health problem in Alabama. Excessive COPD hospitalization risk exists in the Deep South [33], which is a geographic region where many Black Americans experience pernicious health disparities [34]. Nevertheless, the effects of multiple health risks on Alabamians with COPD are complex and not well understood. This study examined variability in discrete risk indicators such as comorbidities, substance use and abuse, and healthcare access among adults with COPD who reside in Alabama, which is a U.S. state with a high burden of COPD. Analyzing structural differences in latent factors based on race was of primary interest to assess the potential for racial disparities in existing health risks.

Results from this study demonstrated that a substantial proportion of Alabamians with COPD are at high risk for having multiple comorbidities. Adults living with COPD are more likely to report having been diagnosed with another chronic condition than adults who do not have COPD [35,36,37]. Of the eight comorbidities considered in this study, the most significant proportion of multi-morbid patients with COPD reported asthma, arthritis, and symptoms of depression. This finding is consistent with national comorbidity estimates in the general COPD patient population [38]. While challenging, healthcare providers across the state of Alabama should work toward standardizing treatment approaches for high-risk Alabamians with COPD, with specific attention given to patients living with specific comorbidities (i.e., asthma, arthritis, and symptoms of depression). These interconnected chronic conditions may contribute to similar symptoms in patients with COPD [39]; thus, Alabamians with COPD, especially those considered to be “high risk,” should be screened and treated for these particular co-occurring conditions that may compound the health complications associated with COPD.

The percentage of patients with COPD who reported arthritis was higher as risk level increased (low risk: ≈50%; intermediate risk: ≈75%; and high risk: ≈95%), meaning that arthritis is more of a concern for patients at high risk for comorbidities as compared to those at low risk. Rheumatoid arthritis (RA) is an increasingly important contributor to COPD [40], which is likely due to inflammatory and autoimmune mechanisms [41]. Consistent with the findings from this study in Alabama, a diagnosis of arthritis is associated with a higher prevalence of COPD across the U.S. population [42]. Individuals with rheumatoid arthritis and COPD have an almost 50% greater risk of COPD-related hospitalization than the general population [43]. The mortality risk is also likely higher among patients with COPD who have rheumatoid arthritis versus those who do not [44,45]. Therefore, healthcare providers should strongly consider assessing COPD and arthritis symptoms together for earlier detection. Dual diagnosis will enable providers to prescribe pulmonary rehabilitation and self-management training interventions that address how to treat both of these conditions simultaneously [42,46]. Given the high prevalence of co-occurring COPD and arthritis in Alabama, future research should seek to understand the effects of arthritis on morbidity and mortality in Alabamians with COPD.

Similar variability in latent risk classes was present for Alabamians with COPD who reported depressive symptoms (low risk: ≈30%; intermediate risk: ≈40%; and high risk: ≈70%). COPD increases the risk of developing anxiety and depression, both of which can negatively affect disease prognosis, most notably by increasing the risk of exacerbations [47]. Alabamians with COPD should be screened and treated for anxiety and depression (if necessary), as patients with these mental health conditions typically show higher levels of shortness of breath than patients without mental disorders [48]. Although some evidence supports pharmacological treatments for anxiety and depression in patients with COPD, only small studies with modest effect sizes support such medications [47]. Primary care physicians should consider referring high-risk Alabamians with COPD with subclinical mental distress to hospital- or clinic-based pulmonary rehabilitation [3] and/or alternative mind–body exercise (i.e., Tai Chi, yoga) programs [49]. One meta-analysis showed that patients with severe symptoms of COPD who participated in self-management interventions focused on mental health *and* symptom management showed more significant improvements in health status with fewer emergency room visits than those who participated in interventions that addressed symptom management alone [50].

About one in five Alabamians with COPD belonged to an intermediate comorbidity risk class with the majority reporting arthritis as well as diabetes and obesity. Although over half of Alabamians with COPD at high risk for comorbidities were obese, the proportion of patients reporting diabetes and obesity within the intermediate risk class was higher than among patients classified as high risk. Other studies report that more than one-third of patients with COPD are obese [51,52]. Interestingly, an “obesity paradox” in COPD [53] may exist, whereby obese patients with COPD may have lower exacerbation frequency [54] and reduced in-hospital mortality caused by exacerbations [55] than their non-obese counterparts. Our analysis demonstrated that obese white Alabamians with COPD were more likely to be at high risk for comorbidities than those who were not obese. This finding contradicted what was found in obese Black Alabamians with COPD who were much less likely to be at high risk for comorbidities. Thus, the impact of obesity on comorbidity risks for patients with COPD may differ based on race, with obese white patients experiencing more health-related challenges caused by unhealthy body weight. Future studies within Alabama should examine racial disparities in hyperglycemia (i.e., high blood sugar) and body mass index values to help understand how race potentially influences the obesity paradox among Alabamians with COPD.

The impact of an asthma diagnosis on comorbidity risk was different between white and Black Alabamians with COPD. White Alabamians who reported asthma with COPD were more likely to be at high risk for comorbidities than whites without asthma. Alternatively, Black Alabamians who reported asthma and COPD were less likely to be at high risk for comorbidities than those without asthma. Almost two-thirds of Black Alabamians with COPD were at intermediate risk for being diagnosed with asthma, which was about six times higher than the proportion of white Alabamians with COPD who reported a diagnosis of asthma. Black adults with COPD or coexisting COPD and asthma use fewer medical services and account for lower medical costs than white adults, which may provide a possible explanation for the racial disparities in outcomes of patients diagnosed with both COPD and asthma [56]. Future research should examine how race influences asthma–COPD overlap (i.e., symptoms of asthma and COPD), including the frequency of dyspnea and exacerbations. Our study also showed that Black Alabamians with COPD, in addition to heart and kidney disease, were much more likely to be at high risk for comorbidities than their counterparts without these comorbidities. A better understanding of the unique, co-occurring symptoms and complications associated with asthma, heart disease, and kidney disease within Black Alabamians with COPD will likely help providers develop personalized treatment strategies.

Almost half of the Alabamians with COPD were at high risk of having limited healthcare access, which is a percentage far higher than adults with COPD living across the U.S. [38]. Over three-quarters of high-risk respondents could not afford medical care, two-thirds were uninsured, and over half did not report having a primary care provider or annual check-up. Lack of healthcare access for Alabamians with COPD is a significant risk factor that statewide public healthcare policymakers should address. Patients with COPD who receive treatment at primary healthcare centers with lung disease-focused clinics experience fewer COPD exacerbations and hospitalizations, and overall, treatment costs are substantially lower [57]. In addition, the role of annual check-ups may influence risk for limited healthcare access differently in white versus Black Alabamians with COPD. In this study, white Alabamians with COPD who did not report an annual check-up were more likely to be at high risk for limited healthcare access than whites who had received their annual check-up.

On the other hand, Black Alabamians with COPD who went for their annual check-up were a bit more likely to be at high risk for limited healthcare access than their counterparts without an annual check-up. Future research should seek to understand the effects of potential racial disparities in access to annual check-ups, as these yearly visits may impact healthcare access and use differently among white and Black Alabamians with COPD. Medicare’s complicated coverage and reimbursement structure pose unique challenges for patients with COPD who might need access to primary and specialty (i.e., pulmonary rehabilitation, respiratory therapy) healthcare services. Gaffney et al. [58], in their longitudinal analysis of National Health Interview Survey data, found that the proportion of patients diagnosed with COPD going without needed medications worsened from 1997 to 2018. Our analysis of BRFSS data from Alabama indicates that ongoing barriers to available pulmonary rehabilitation and respiratory therapies may be preventing effective disease management for high-risk Alabamians with COPD.

This study also suggests a noteworthy deficiency in vaccinations among Alabamians with COPD. Over 80% of high-risk Alabamians with COPD did not receive their annual flu shot, while slightly more than 60% reported no vaccination against pneumonia. Other available data supports that Alabamians with COPD have a lower than average influenza vaccination rate [9]. In this study, white Alabamians with COPD who received their annual flu shot and pneumonia vaccine were at markedly low risk for limited healthcare access compared to white patients who did not receive these vaccines. However, low vaccination rates remain problematic for Alabamians living with COPD because they are particularly vulnerable to influenza, which can cause otherwise preventable breathing exacerbations [59]. The target *Healthy People 2030* recommendation for flu vaccination is 70% for the general population [60], which is a percentage more than 25% higher than the current flu vaccine rate of Alabamians with COPD [9]. Despite positive benefit-to-risk ratios for seasonal influenza vaccination in patients with COPD [59,61], the proportion of Alabamians with COPD who receive their flu vaccine is wholly inadequate. Future research should seek to understand the perceived benefits and barriers toward preventive vaccines among Alabamians living with COPD. Making vaccination for these communicable diseases a statewide priority will help ensure that Alabamians with COPD are protected against the adverse respiratory effects of influenza and pneumonia.

The COVID-19 pandemic may very well have a substantial impact on the health risk indicators considered in this study. With the rapid emergence of the COVID-19 pandemic, there is yet another, even higher, vaccination priority for adults living with COPD. COPD patients are at increased risk of severe pneumonia and other poor health outcomes when they contract COVID-19; therefore, future research on COVID-19 in COPD should continue to examine how the burden of disease and clinical manifestations in COPD patients differ from the general population [62]. The COVID-19 pandemic has also shed substantial light on noteworthy health and healthcare disparities for specific racial/ethnic minority groups in the U.S. [63,64]. Being a member of certain racial/ethnic minority groups with low socioeconomic status negatively impacts healthcare access and utilization [63]. In this study, Black Alabamians without health insurance were much more likely to be at high risk for limited healthcare access when compared to insured Black Alabamians with COPD. The proportion of high-risk, uninsured Black Alabamians with COPD greatly exceeded the proportion of high-risk, uninsured white Alabamians with COPD. Furthermore, almost 80% of high-risk Black Alabamians with COPD reported cost as preventing access to healthcare, which could undoubtedly perpetuate hesitancy toward seeking medical care when needed.

Future studies should pay special attention to the vaccination status of minority groups with COPD, especially within Alabama. Perceived barriers to vaccination may be similar to those that preclude Black Alabamians from being prescribed treatments such as pulmonary rehabilitation [64]. Other studies have shown that factors limiting access to healthcare include financial costs, lack of transportation, and lack of a formal or informal caregiver [62], which can be especially problematic for Blacks with COPD who have elevated comorbidity risks [63]. Narrowing existing disparities will inevitably involve improving access to affordable healthcare and medication coverage [63]. Based on the results of this study, it will be essential to evaluate the impact of post-COVID-19 infection disability in COPD patients from racial/ethnic minority groups. Subsequent studies can help determine what resources will be required to adequately support care coordination for COPD patients after COVID-19, especially in isolated and resource-limited settings [62]. Interestingly, only 5% of Alabamians with COPD belonged to the high-risk class for substance use and abuse; most were at low risk for engaging in substance abuse. Over 80% of Alabamians with COPD were classified into the low-risk substance use class. Still, about one-third of these low-risk Alabamians identified as current smokers. Most U.S. adults with COPD smoke every day and binge drink or drink heavily at least once over the past 30 days [38]. Nearly half (42.69%) of Central Appalachians with COPD were at high risk for substance use due to daily smoking and heavy alcohol use [65]. While the majority of Alabamians with COPD do not seem to be engaging in these types of high-risk substance use and abuse behaviors, it is worth noting that Blacks diagnosed with COPD are more likely to experience the adverse health effects of tobacco smoke due to biological, socioeconomic, and cultural factors [12,66,67,68].

### Limitations

This study is not without limitations. The secondary analysis included multiple years of Alabama BRFSS data. The BRFSS is based on telephone survey data that includes unvalidated self-reports affected by recall bias. Given the limited number and standardized content of BRFSS items, future research should consider incorporating other variables (e.g., interpersonal relationships, community/social ties) above and beyond the individual and health system indicators assessed in this study. Such analyses should consider county-level data from Alabama that enable more localized comparisons across the state. Alternatively, to place our preliminary findings in an appropriate context, future analyses should compare differences in health risk indicators between Alabamians diagnosed with COPD and those without COPD.

Limitations of the LCM approach used to analyze BRFSS data include the relatively limited theoretical support for the three domains considered in this study. For example, smaller size classes identified in this study may represent outliers in the BRFSS data. In addition, although BRFSS is a telephone-based survey, one strength of this study is that it includes an extensive sample of respondents randomly drawn from Alabama rather than from convenience samples of patients with COPD. As a result of this, the generalizability of our results represents a positive characteristic of this population-level secondary data analysis.

In addition, this study coded all Black Alabamians with COPD homogenously according to the BRFSS item on race, which ignores variations in socioeconomic status, tobacco smoking behavior, access to healthcare, and health insurance coverage among Black Americans. As suggested elsewhere [12], to better capture racial diversity, future epidemiological and observational analyses should consider data from foreign-born and U.S.-born black individuals, including place of birth, country of origin, and socioeconomic status.

## 5. Conclusions

Our analysis of 2015–2019 BRFSS data from Alabama showed that almost half of Alabamians with COPD were at high risk for having limited healthcare access. Moreover, over two-thirds of Black Alabamians with COPD were at high risk of being uninsured, exceeding the proportion of high-risk white Alabamians with COPD. In addition, the vast majority of Alabamians with COPD did not receive essential vaccines for influenza and pneumonia, both of which can have very adverse health consequences for vulnerable adults with COPD.

Findings from this multi-year secondary analysis have important practice implications for informing statewide policies and interventions that will enable Alabamians with COPD to mitigate the effects of comorbidities and inadequate healthcare access. Disparities in health risk profiles should be addressed across the state to improve the health status of Alabamians with COPD. In particular, greater focus should be placed on racial/ethnic subgroups with limited healthcare access. As healthcare systems work to improve the care of COPD patients across Alabama, existing disparities must be quantified to assure the equitable implementation of risk mitigation interventions for all racial and ethnic groups. Data-based policies and interventions may reduce the impact of specific personal and health system access risk factors that disproportionately affect the health of Alabamians living with COPD.

## Figures and Tables

**Table 1:** Classification frequencies of comorbidity risks for Black and white Alabamians with COPD, BRFSS, 2015–2019.

Comorbidities	White			Black		
Asthma	Yes n (%)	No n (%)	*p*-value	Yes n (%)	No n (%)	*p*-value
Low Risk	368 (27.88)	952 (72.12)	<0.0001	133 (30.09)	309 (69.91)	<0.0001
Intermediate Risk	41 (10.85)	337 (89.15)		255 (63.91)	144 (36.09)	
High Risk	775 (63.27)	450 (36.73)		7 (25.00)	21 (75)	
Diabetes	Yes n (%)	No n (%)	*p*-value	Yes n (%)	No n (%)	*p*-value
Low Risk	0 (0.00)	1324 (100)	<0.0001	60 (13.61)	381 (86.39)	<0.0001
Intermediate Risk	286 (75.46)	93 (24.54)		217 (55.08)	177 (44.92)	
High Risk	519 (42.51)	702 (57.49)		14 (51.85)	13 (48.15)	
Obesity	Yes n (%)	No n (%)	*p*-value	Yes n (%)	No n (%)	*p*-value
Low Risk	224 (17.72)	1040 (82.28)	<0.0001	133 (32.44)	277 (67.56)	<0.0001
Intermediate Risk	166 (45.60)	198 (54.40)		301 (80.48)	73 (19.52)	
High Risk	678 (57.56)	500 (42.44)		2 (7.69)	24 (92.31)	
Cardiovascular Disease (CVD)	Yes n (%)	No n (%)	*p*-value	Yes n (%)	No n (%)	*p*-value
Low Risk	68 (5.23)	1233 (94.77)	<0.0001	20 (4.59)	416 (95.41)	<0.0001
Intermediate Risk	148 (39.57)	226 (60.43)		103 (26.21)	290 (73.79)	
High Risk	423 (35.67)	763 (64.33)		23 (82.14)	5 (17.86)	
Arthritis	Yes n (%)	No n (%)	*p*-value	Yes n (%)	No n (%)	*p*-value
Low Risk	704 (53.09)	622 (46.91)	<0.0001	181 (41.42)	256 (58.58)	<0.0001
Intermediate Risk	168 (44.09)	213 (55.91)		376 (94.24)	23 (5.76)	
High Risk	1192 (97.47)	31 (2.53)		24 (85.71)	4 (14.29)	
Stroke	Yes n (%)	No n (%)	*p*-value	Yes n (%)	No n (%)	*p*-value
Low Risk	66 (4.97)	1261 (95.03)	<0.0001	25 (5.64)	418 (94.36)	<0.0001
Intermediate Risk	29 (7.69)	348 (92.31)		86 (21.50)	314 (78.50)	
High Risk	345 (28.21)	878 (71.79)		15 (53.57)	13 (46.43)	
Depressive Disorder	Yes n (%)	No n (%)	*p*-value	Yes n (%)	No n (%)	*p*-value
Low Risk	413 (31.24)	909 (68.76)	<0.0001	97 (22.25)	339 (77.75)	<0.0001
Intermediate Risk	42 (11.02)	339 (88.98)		232 (58.00)	168 (42.00)	
High Risk	855 (70.31)	361 (29.69)		16 (57.14)	12 (42.86)	
Chronic Kidney Disease	Yes n (%)	No n (%)	*p*-value	Yes n (%)	No n (%)	*p*-value
Low Risk	16 (1.20)	1313 (98.80)	<0.0001	10 (2.26)	432 (97.74)	<0.0001
Intermediate Risk	41 (10.88)	336 (89.12)		43 (10.72)	358 (89.28)	
High Risk	214 (17.60)	1002 (82.40)		28 (100.00)	0 (0.00)	

**Table 2:** Classification frequencies of limited healthcare indicators for Black and white Alabamians with COPD, BRFSS, 2015–2019.

Healthcare Indicator	White			Black		
Access to Health Insurance	Yes n (%)	No n (%)	*p*-value	Yes n (%)	No n (%)	*p*-value
Low Risk	1360 (99.34)	9 (0.66)	<0.0001	186 (100)	0 (0)	<0.0001
Intermediate Risk	1211 (98.06)	24 (1.94)		575 (96.48)	21 (3.52)	
High Risk	135 (40.30)	200 (59.70)		5 (5.56)	85 (94.44)	
Primary Care Physician	Yes n (%)	No n (%)	*p*-value	Yes n (%)	No n (%)	*p*-value
Low Risk	28 (2.05)	1341 (97.95)	<0.0001	31 (16.67)	155 (83.33)	<0.0001
Intermediate Risk	184 (55.09)	1208 (97.66)		11 (1.84)	586 (98.16)	
High Risk	29 (2.34)	150 (44.91)		41 (45.56)	49 (54.44)	
Afford Medical Costs	Yes n (%)	No n (%)	*p*-value	Yes n (%)	No n (%)	*p*-value
Low Risk	1198 (87.51)	171 (12.49)	<0.0001	106 (56.99)	80 (43.01)	<0.0001
Intermediate Risk	1030 (83.40)	205 (16.60)		494 (83.16)	100 (16.84)	
High Risk	70 (20.96)	264 (79.04)		11 (12.22)	79 (87.78)	
Check-Up in Past Year	Yes n (%)	No n (%)	*p*-value	Yes n (%)	No n (%)	*p*-value
Low Risk	1253 (92.47)	102 (7.53)	<0.0001	138 (75)	46 (25)	<0.0001
Intermediate Risk	1100 (90.53)	115 (9.47)		579 (97.64)	14 (2.36)	
High Risk	105 (31.91)	224 (68.09)		47 (52.81)	42 (47.19)	
Annual Flu Shot	Yes n (%)	No n (%)	*p*-value	Yes n (%)	No n (%)	*p*-value
Low Risk	1369 (100)	0 (0)	<0.0001	5 (2.87)	169 (97.13)	<0.0001
Intermediate Risk	142 (13.15)	938 (86.85)		336 (62.69)	200 (37.31)	
High Risk	41 (13.23)	269 (86.77)		8 (9.88)	73 (90.12)	
Pneumococcal Vaccine	Yes n (%)	No n (%)	*p*-value	Yes n (%)	No n (%)	*p*-value
Low Risk	778 (100)	0 (0)	<0.0001	12 (9.30)	117 (90.70)	<0.0001
Intermediate Risk	320 (45.58)	382 (54.42)		246 (84.54)	45 (15.46)	
High Risk	67 (38.29)	108 (61.71)		16 (35.56)	29 (64.44)	

## Data Availability

The data can be downloaded from the CDC website: https://www.cdc.gov/brfss/annual_data/annual_2019.html (accessed on 16 April 2021).
